# Comprehensive assessment of HF-rTMS treatment mechanism for post-stroke dysphagia in rats by integration of fecal metabolomics and 16S rRNA sequencing

**DOI:** 10.3389/fcimb.2024.1373737

**Published:** 2024-04-15

**Authors:** Fei Zhao, Jiemei Chen, Yilong Shan, Jiena Hong, Qiuping Ye, Yong Dai, Jiahui Hu, Jiantao Zhang, Chao Li, Hongmei Wen

**Affiliations:** ^1^ The Third Affiliated Hospital of Sun Yat-sen University, Department of Rehabilitation Medicine, Guangzhou, Guangdong, China; ^2^ Guangzhou University of Chinese Medicine, Clinical Medical College of Acupuncture Moxibustion and Rehabilitation, Guangzhou, Guangdong, China

**Keywords:** stroke, middle cerebral artery, rats, dysphagia, gut microbiota, metabolite profiles, HF-rTMS

## Abstract

**Background:**

The mechanism by which high-frequency repetitive transcranial magnetic stimulation (HF-rTMS) improves swallowing function by regulating intestinal flora remains unexplored. We aimed to evaluate this using fecal metabolomics and 16S rRNA sequencing.

**Methods:**

A Post-stroke dysphagia (PSD) rat model was established by middle cerebral artery occlusion. The magnetic stimulation group received HF-rTMS from the 7th day post-operation up to 14th day post-surgery. Swallowing function was assessed using a videofluoroscopic swallowing study (VFSS). Hematoxylin-eosin (H&E) staining was used to assess histopathological changes in the intestinal tissue. Intestinal flora levels were evaluated by sequencing the 16S rRNA V3-V4 region. Metabolite changes within the intestinal flora were evaluated by fecal metabolomics using liquid chromatography-tandem mass spectrometry.

**Results:**

VFSS showed that the bolus area and pharyngeal bolus speed were significantly decreased in PSD rats, while the bolus area increased and pharyngeal transit time decreased after HF-rTMS administration (p < 0.05). In the PSD groups, H&E staining revealed damaged surface epithelial cells and disrupted cryptal glands, whereas HF-rTMS reinforced the integrity of the intestinal epithelial cells. 16S rRNA sequencing indicated that PSD can disturb the intestinal flora and its associated metabolites, whereas HF-rTMS can significantly regulate the composition of the intestinal microflora. Firmicutes and Lactobacillus abundances were lower in the PSD group than in the baseline group at the phylum and genus levels, respectively; however, both increased after HF-rTMS administration. Levels of ceramides (Cer), free fatty acids (FA), phosphatidylethanolamine (PE), triacylglycerol (TAG), and sulfoquinovosyl diacylglycerol were increased in the PSD group. The Cer, FA, and DG levels decreased after HF-rTMS treatment, whereas the TAG levels increased. Peptococcaceae was negatively correlated with Cer, Streptococcus was negatively correlated with DG, and Acutalibacter was positively correlated with FA and Cer. However, these changes were effectively restored by HF-rTMS, resulting in recovery from dysphagia.

**Conclusion:**

These findings suggest a synergistic role for the gut microbiota and fecal metabolites in the development of PSD and the therapeutic mechanisms underlying HF-rTMS.

## Introduction

Post-stroke dysphagia (PSD) has emerged as a prevalent and consequential complication subsequent to an episode of stroke, with a prevalence of more than 50% (Bonilha et al., 2014; Zhang et al., 2021). Moreover, more than 10% of patients have persistent symptoms of dysphagia until six months after onset (Dawson et al., 2016). PSD is associated with a risk of aspiration pneumonia, malnutrition, dehydration, and death (Shigematsu and Fujishima, 2015). Therefore, there is an urgent need to develop effective therapeutic methods for reducing these risks. Routine therapies such as postural substitution and adjustment of food texture do not have satisfactory effectiveness (Reyes-Torres et al., 2019; Lin et al., 2021). However, recent studies have reported that noninvasive brain stimulation such as transcranial magnetic stimulation (TMS) is effective in improving swallow function (Chiang et al., 2019; Du et al., 2022; Hammad et al., 2022).

A meta-analysis unveiled a heightened effect size in dysphagia rehabilitation within the high-frequency rTMS (HF-rTMS) subgroup, surpassing that observed in the low-frequency rTMS (LF-rTMS) subgroup (Liao et al., 2017). In line with this, Khedr et al. (Khedr and Abo-Elfetoh, 2009) administered HF-rTMS (3 Hz) treatment to dysphagia patients, observing superior clinical outcomes in the treatment group compared to the sham group. Regarding to the stimulation site of HF-rTMS, Park et al. (Park et al., 2013) found that when 5Hz HF-rTMS applied to the contralateral cerebral hemisphere of patients with PSD, the swallowing function of the treatment group was significantly better than that of the sham stimulation group. Cheng et al. (Cheng et al., 2015) also proved that HF-rTMS applied over the tongue region of the motor cortex of the unaffected hemisphere improves the swallowing performance in stroke patients with chronic dysphagia. These studies focus on the mechanism of HF-rTMS at the central level. It is noteworthy that a previous study has suggested a potential impact of ischemic stroke on the gut microbiota, resulting in increased intestinal permeability and activation of the intestinal immune system. This, in turn, exacerbates ischemia-reperfusion injury through the brain-gut axis. Conversely, fecal microbiota transplantation is neuroprotective after stroke (Singh et al., 2016). Furthermore, gut microbiota metabolites can affect the physiological status of the host, both within the gut and after entering the bloodstream, by acting as a bridge between the microbiome and host. For example, short-chain fatty acids (SCFAs) may affect post-stroke outcomes via local and systemic inflammation and other pathways (Benakis et al., 2020). A recent study suggested that stimulation of the dorsolateral prefrontal cortex by HF-rTMS modifies brain-gut interactions in humans, for example, by modifying fine contractions of the gastrointestinal tract (Aizawa et al., 2021). Similarly, in rat model of depression, HF-rTMS (10 Hz) primarily contributes to the increased abundance of Firmicutes (Seewoo et al., 2022). These studies suggest that HF-rTMS can affect intestinal flora, but studies on its role in PSD have not been reported. In this study, we hypothesized that PSD could influence the composition of gut microbial communities and metabolites, which may be modified by HF-rTMS, resulting in improved swallowing function. To verify this, we investigated these mechanisms by integrating fecal metabolomics and 16S rRNA sequencing at the rat level.

## Materials and methods

### Animals

Thirty-six male Sprague-Dawley (SD) rats (aged 6-8 weeks) were purchased from the Guangdong Medical Laboratory Animal Center (Guangzhou, China). The rats were housed under a 12 h light-dark cycle (lights on 07:00) with controlled temperature (20-26°C), relative humidity (40-70%), and had free access to food and water. The rats were randomly assigned to experimental groups. Rats were allowed to acclimate for five days before the experiments were started. All animal experiments were approved by the Animal Care Committee of the South China Agricultural University (Guangzhou, China. Approval number NO.2020d030) and were performed in accordance with the ARRIVE Guidelines.

### PSD models and experimental grouping

**Figure 1:**
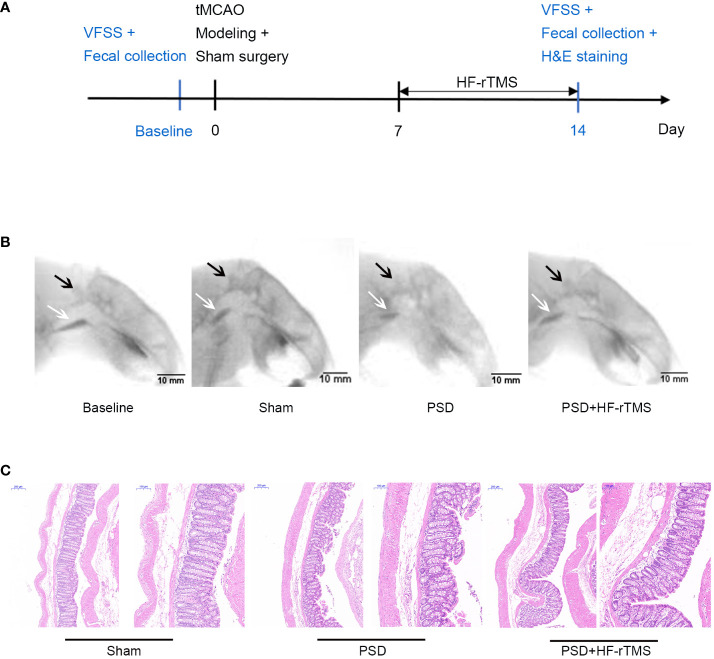
Schematics of experimental design **(A)**, representative radiographic image from rats undergoing videofluorography **(B)**, and Hematoxylin-eosin (H&E) Staining of the colon tissue **(C)**. (scale bar = 200 and 100 μm). VFSS, Videofluoroscopy swallowing study; C2, the second cervical vertebra; black arrow, C2; white arrow, bolus.

The rat PSD model was established using transient middle cerebral artery occlusion (tMCAO). Thirty SD rats were randomly selected for left transient middle cerebral artery occlusion. After administering intraperitoneal anesthesia with 0.4% pentobarbital (40 mg/kg), the rats were fixed on a flat plate, the hair on the neck region was removed and disinfected with iodine. An incision (10 mm long) was made on the left side of the neck to expose the common carotid artery (CCA) and external carotid artery (ECA). The proximal ends of the left CCA and the ECA were ligated. A monofilament nylon line (50 mm in length; 0.26 mm in diameter) coated with 5-mm silica gel in the distal segment was used. Monofilament lines were inserted into the internal carotid artery (ICA) through a left CCA incision. The line was then inserted at a depth of 18-20 mm (beginning at the carotid bifurcation) to block the left middle cerebral artery. Finally, the skin was sutured and the rats were placed on a thermal insulation blanket. After 90 min of ischemia, the monofilament lines were carefully extracted from the ICA to induce reperfusion. Seventeen rats survived beyond 7 days postoperatively without mortality. Based on Videofluoroscopy swallowing study (VFSS) assessments, 12 of the 17 surviving rats that displayed dysphagia were randomly divided into two groups: the PSD group (n = 6) and the PSD+HF-rTMS group (n = 6). During the VFSS enrollment assessment of the 12 model rats, stroke severity was evaluated using the Longa scale. The scores obtained ranged from 1 to 2 points, indicating uniformity in neurological function severity among the rats (Zhong et al., 2022). Additionally, the sham operation group (n = 6) of SD rats was subjected to the same protocol without monofilament insertion. The detailed experimental schedule is shown in **Figure 1A**.

### HF-rTMS treatment

**Figure 2:**
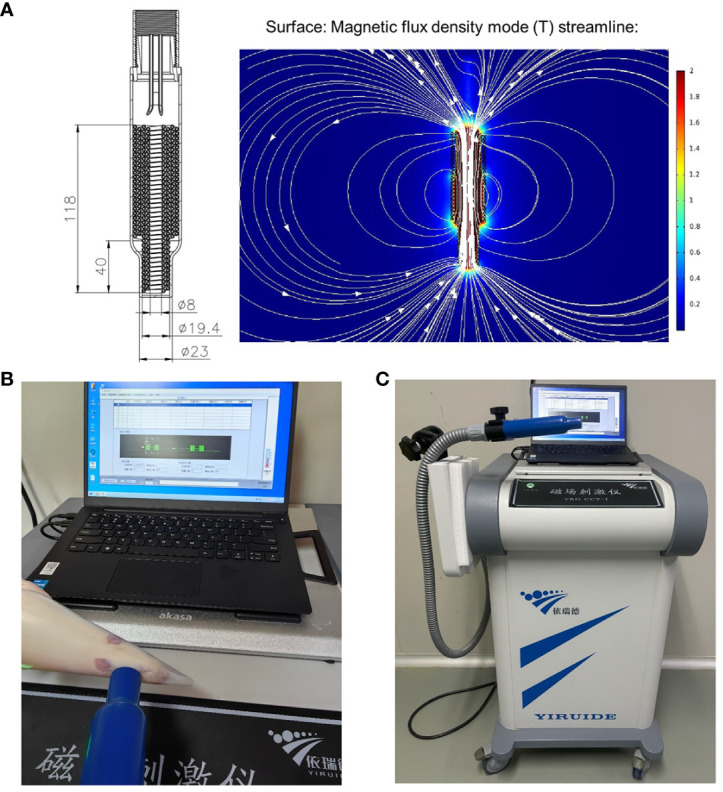
The animal special coil mechanism diagram **(A)** and pictures of rTMS administered in animal model **(B, C)**.

HF-rTMS was administered using a customized stimulator (YRDCCI, Wuhan, China). On the 7^th^ day post-operation, the magnetic stimulation group received rTMS treatment, which was continued until the 14^th^ day post-surgery. A round prototype coil (23mm diameter with 3.5T peak magnetic welds) was positioned perpendicular to the cortical surface projection area of the right primary motor cortex (M1). TMS-induced electric field modeled in the animal’s brain is 140 V/m. The treatment protocol was as follows: stimulation for 5 s and then, rest for 60 s (repeated 10 times); stimulation intensity, 33% of the maximum stimulator output; stimulation frequency, 10Hz. The animal special coil mechanism diagram and pictures of rTMS administered in animal models in **Figures 2A-C**.

### Videofluoroscopy swallowing study (VFSS) assessments

The rats underwent VFSS on the day before surgery (baseline) and 2 weeks after surgery (day 14). The rats were fasted for 12 h before the VFSS assessments. A mixture of experimental rat breeding feed and iohexol (1:1) was placed on a platform in a transparent plastic box. During ad libitum feeding of the feed mixture, videos were obtained at 30 fps using swallowing radiography equipment (digital fluoroscopic X-ray machine PLD8100C, Zhuhai, China). Only swallows with clear sagittal views were analyzed. The mean fluoroscopic results for each rat were calculated using 8-10 swallows.

ImageJ software (National Institutes of Health, Bethesda, MD) was used to assess swallowing function, including 1) bolus area (mm^2^): bolus size measured after swallow initiation and before the head of the bolus passed through the second cervical vertebra (C2); 2) Pharyngeal Transit Time (PTT, s): initiated on bolus passing through the posterior oropharynx and ended on it completely passing through C2, while the position was near the upper esophageal sphincter; 3) pharyngeal bolus speed (mm/s): the maximum speed at which the head of the bolus traveled from the initiation point to C2; and 4) inter-swallow interval (s): time interval between two successive swallows during uninterrupted eating (Russell et al., 2013; Welby et al., 2021).

### Hematoxylin–eosin (H&E) staining of intestinal tissue

All rats were sacrificed on the 14^th^ days after surgery. The Intestinal tissues (3 samples of each group) were placed in 4% paraformaldehyde for 24 h, mounted with gelatin, and then, embedded in paraffin. The tissues were cut into 10 μm slices; after which the sections were deparaffinized and stained with histopathological and eosin (Servicebio, G1076).

### Fecal collection

We collected feces early in the morning after lights were turned on at the housing facility. The rats were temporarily placed individually in empty autoclaved cages and allowed to defecate. Approximately two fecal pellets were collected aseptically from each rat and placed in a sterile 2-mL tube. Fecal samples from four rats were collected from each group and stored at -80.0°C refrigerator. The time point for fecal sample collection coincided with that of the VFSS assessments.

### DNA extraction and 16S rRNA gene sequencing of fecal samples

The stool samples of each group were removed from the -80°C refrigerator. Total genomic DNA from stool samples was extracted using the CTAB/SDS method, and its concentration and purity were monitored using 1% agarose gels according to the concentration. The DNA was diluted to 1 ng/µL using sterile water. Primers were designed to amplify the variable V3-V4 regions of the 16S rRNA gene. PCRs was performed with 15 mL of Phusion^®^ High-Fidelity PCR Master Mix (New England Biolabs, USA), 0.2 mM forward and reverse primers, and approximately 10 ng of template DNA. The initial denaturation temperature was set at 98°C for 1 min, followed by denaturation at 98°C for 10 s (30 cycles), annealing at 50°C for 30 s, extension at 72°C for 1 min, and final elongation at 72°C for 8 min and storage at 10°C. PCR products were electrophoresed on a 2% agarose gel and purified using the Qiagen Gel Extraction Kit (Qiagen, Germany). The TruSeq DNA PCR-Free Sample Preparation Kit (Illumina, USA) was used to construct the library, which was assessed using an Agilent Bioanalyzer 2100 system. After the library was qualified, the Illumina NovaSeq was used for 16S rRNA V3-V4 sequencing. We spliced and filtered the original data to remove contaminated data and to obtain accurate and reliable data.

### Evaluation of microbiome composition based on 16S rRNA sequences and informatics analysis

Uparse software was used to cluster clean reads from all samples, and sequence clustering was converted into OTUs) by default with 97% identity. Species annotation analysis was performed using the Silva Database based on the Mothur algorithm to evaluate the differences and community composition of the dominant species in different samples. Raw data were analyzed using BioTree Biotechnology Co., Ltd. (Shanghai, China). Alpha and beta diversities were calculated using the breakaway method implemented in Quantitative Insights into Microbial Ecology, version 2.0. Finally, linear discriminant analysis effect size and linear discriminant analysis were used to identify the dominant bacterial taxa in the different groups of rats.

### Liquid chromatography–mass spectrometry (LC-MS/MS) based metabolomics

A total of 25 mg of fecal sample was weighed and placed in an EP tube. Then, 200 μL of water and 480 μL of extract solution (MTBE: MeOH = 5:1) were added sequentially. After vortexing for 30 s, the samples were homogenized for 4 min at 35 Hz and sonicated for 5 min in an ice-water bath. Homogenization and sonication cycles were repeated three times. Next, the samples were incubated at -40°C for 1 h and centrifuged at 3000 rpm for 15 min at 4°C. The supernatant (300 μL) was transferred to a fresh tube and dried under vacuum at 37°C. Then, The dried samples were reconstituted in 200 μL of 50% methanol in dichloromethane by sonication for 10 min in ice water bath. The mixture was then centrifuged at 13000 rpm for 15 min at 4°C, and 150 μL of the supernatant was collected and stored in a refrigerator at -20°C until LC-MS/MS analysis was performed.

LC-MS/MS analysis was performed using a UHPLC system (Vanquish, Thermo Fisher Scientific) with a UPLC HSS T3 column (2.1 mm × 100 mm; 1.8 μm) coupled with a Q Exactive HFX mass spectrometer (Orbitrap MS, Thermo). The Mobile phase A consisted of 40% water, 60% acetonitrile, and 10 mM ammonium formate. The Mobile phase B consisted of 10% acetonitrile and 90% isopropanol, which was added with 50 mL of 10 mmol/L ammonium formate for every 1000 mL mixed solvent. Analysis was performed using an elution gradient. The column temperature was 55°C. The autosampler temperature was maintained at 4°C. A Triple TOF 5600 + mass spectrometer (AB SCIEX, USA) was used for the electrospray ionization (ESI) positive and negative ion mass spectrometry analyses. ESI conditions: ion source gas 1:60, ion source gas 2:60, temperature 600°C, voltage ± 5500 V in positive and negative modes, mass scanning range m/z:60-1000 Da, first-level scanning range 25-1000 Da, scanning time 0.2 s, and high-sensitivity mode was used. For the second mass spectrometry, the delustering voltages of the positive and negative modes were ± 60 V and the high collision energy was (35 ± 15) eV.

### Screening and identification of main differential metabolites

The raw data files were converted to files in the mzXML format using the ‘msconvert’ program from ProteoWizard. The CentWave algorithm in XCMS was used for peak detection, extraction, alignment, and integration; the minfrac for annotation was set at 0.5, and the cutoff for annotation was set at 0.3. Supervised orthogonal projections to latent structure discriminant analysis (OPLS-DA) was used to screen for the differential metabolites. Variable importance in projection (VIP) ≥ 1.0 and absolute fold change ≥ 3.0, were used as criteria for differential metabolite selection.

### Statistical analyses

Data are presented as mean ± standard error of the mean (SEM). One-way ANOVA variance was performed to compare the continuous variables across multiple groups. All statistical analyses and correlation graphs were generated using GraphPad Prism 8 (GraphPad Software, San Diego, CA, United States).

Three different parameters (observed operational taxonomic units (OTUs) and Shannon and Simpson indices) were used to assess the alpha diversity. Beta diversity between samples was assessed using principal coordinate analysis (PCoA) and permutational multivariate analysis of variance (PERMANOVA). Phylogenetic Investigation of Communities by Reconstruction of Unobserved States (PICRUSt) was utilized to explore differences in the Kyoto Encyclopedia of Genes and Genomes (KEGG) pathways in bacterial taxa between groups. MetaboAnalyst 5.0 (Xia Lab, McGill University, Canada) was used to analyze metabolomics data, including a pathway analysis overview. The association between the main differential metabolites and gut butyrate-producing bacteria was assessed using Spearman’s rank correlation and correlation heat maps were drawn using R Studio (version 3.6.1, Boston, Massachusetts, USA). *p*-value < 0.05.

## Results

### VFSS analysis

A total of 24 recorded swallowing videos (six rats at baseline and six rats each from the PSD, PSD+HF-rTMS, and Sham groups on the 14^th^ postoperative day) were manually analyzed. As shown in **Table 1**, compared with the baseline group, the bolus area and pharyngeal bolus speed were significantly decreased in the PSD group, and there were significant increases in the bolus area and decreases in PTT following HF-rTMS intervention (*p* < 0.05). Representative radiographic images of the rats undergoing videofluorography are shown in **Figure 1B**. According to these results, middle cerebral artery occlusion can result in dysphagia, which can be mitigated by HF-rTMS intervention.

**Table 1:** Results of VFSS swallowing assessment.

	Baseline group (n=6)	Sham group (n=6)	PSD group (n=6)	PSD+ HF-rTMS group (n=6)
Bolus Area (mm^2^)	20.73 ± 1.04	18.27 ± 1.24	8.45 ± 0.61^*^	18.65 ± 0.77^†^
Pharyngeal Transit Time (PTT, s)	1.37 ± 0.17	1.17 ± 0.14	1.67 ± 0.28	1.03 ± 0.17^†^
Pharyngeal Bolus Speed (mm/s)	375.68 ± 35.81	366.06 ± 14.35	297.05 ± 21.20^*^	344.09 ± 11.30
Inter-Swallow Interval (ISI, s)	1.59 ± 0.13	1.69 ± 0.28	2.7 ± 0.30	2.45 ± 0.65

Data were presented as the mean ± SEM. VFSS, Videofluoroscopy swallowing study.

^*^: Compared with Baseline group, p < 0.05.

^†^: Compared with PSD group, p < 0.05.

### Histopathological changes of intestinal tissue

H&E staining revealed that the colon epithelial cells of rats in the sham group had intact structures and were tightly arranged. In contrast, the rats in the PSD group exhibited damaged surface epithelial cells and disrupted cryptal glands. However, in the PSD+HF-rTMS group, there was a reduction in damage, thickening of the intestinal mucous layer, and recovery of the appearance of the surface epithelium (**Figure 1C**). These findings suggest that HF-rTMS intervention may have a positive restorative effect on intestinal tissues in rats with PSD, thereby further reinforcing intestinal epithelial cell integrity.

### Gut microbial profiles

The rarefaction, Shannon-Wiener, and rank-abundance curves in the three groups tended to be flat or plateaued, thus demonstrating satisfactory sequencing depth (**Supplementary Figure 1**).

**Figure 3:**
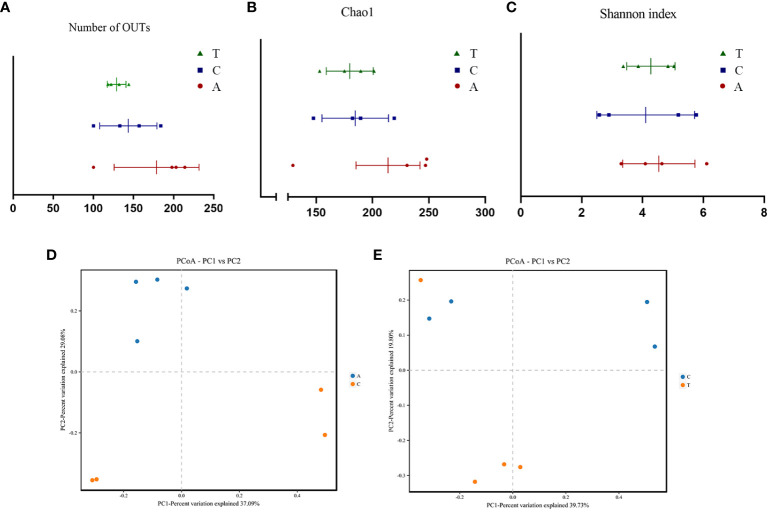
The α-diversity and PCoA analysis of fecal samples based on 16S rRNA gene sequences. Community Richness was quantified based on the number of observed species **(A)** and Chao1 index **(B)**, and community diversity was characterized using the Simpson index **(C)**. Neither richness or diversity differed significantly among the three groups. PCoA analysis between Baseline and PSD group **(D)** and PSD and PSD+HF-rTMS group **(E)**, based on Bray-Curtis distances at the OTU level. OTU, operational taxonomic unit; PCoA, principal coordinates analysis; A, Baseline group; C, PSD group; T, PSD+HF-rTMS group.

Alpha diversity analysis revealed no significant differences in the observed OTUs and Chao1 and Shannon indices among the baseline, PSD, and HF-rTMS groups (**Figures 3A-C**). The PCoA and PERMANOVA analysis of variance for beta diversity revealed a significant difference in the composition and abundance of the gut microbiota between the baseline and PSD groups. (Bray-Curtis *p* < 0.05; R^2^ = 0.287) (**Figure 3D**). The PCoA and PERMANOVA analyses did not show significant differences in the composition and abundance of the gut microbiota between the PSD+HF-rTMS group and PSD group (Bray-Curtis *p* > 0.05, R^2^ = 0.173) (**Figure 3E**).

### Effect of PSD and HF-rTMS on gut microbiota

**Figure 4:**
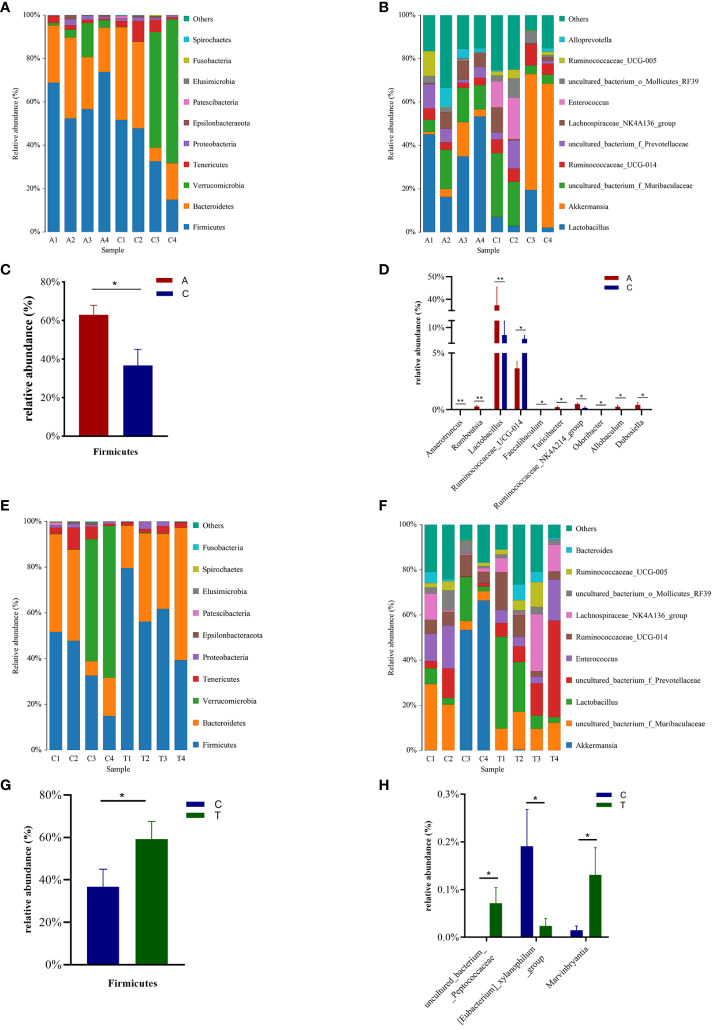
The distributions of the predominant bacteria among the Baseline, tMCAO, and PSD+HF-rTMS group rats. Significantly differential microbes are shown as mean ± SEM. Results at the phylum level **(A, C, E, G)**. Results at the genus level **(B, D, F, H)**. A, Baseline group; C, PSD group; T, PSD+HF-rTMS group; SEM, Standard error of mean; * *p* < 0.05, ** *p* < 0.01 (one-way ANOVA).

Taxon-dependent analysis revealed the top 10 phyla in the Baseline, PSD, and PSD+HF-rTMS groups, with *Firmicutes*, *Bacteroidetes*, *Verrucomicrobia*, *Tenericutes*, and *Proteobacteria* being the most dominant phyla (**Figures 4A, E**). Although no significant differences in the gut microbiota composition at the phylum level were observed among the three groups, the abundance of the phylum *Firmicutes* was significantly lower in the PSD group than in the baseline group (*p* < 0.05) (**Figure 4C**), and the abundance of *Verrucomicrobia* showed an increasing trend without statistical significance (*p* > 0.05). Notably, the abundance of *Firmicutes* increased after HF-rTMS treatment in the PSD+HF-rTMS group (*p* < 0.05) (**Figure 4G**).

The most relative abundant genera (> 1.0%) in the three groups are shown in **Figures 4B, F**. At the genus level, the proportion of *Lactobacillus*, *Anaerotruncus*, *Romboutsia*, *Turicibacter*, *Ruminococcaceae_NK4A214_group*, *Allobaculum*, *Dubosiella*, and *Faecalibaculum* (*p* < 0.05) decreased, whereas those of *Ruminococcaceae_UCG-014* and *Odoribacter* increased in the PSD group (*p* < 0.05) (**Figure 4D**). In the PSD+HF-rTMS group, the relative abundance of *Lactobacillus* and *uncultured_bacterium_f_Prevotellaceae* showed an increasing trend; however, the difference was not statistically significant. Although the relative abundance was < 1.0%, the proportion of *uncultured_bacterium_Peptococcaceae* and *Marvinbryantia* increased, whereas the abundance of *[Eubacterium]_xylanophilum_group* decreased (*p* < 0.05) (**Figure 4H**).

### Identification of group gut microbiota biomarkers

**Figure 5:**
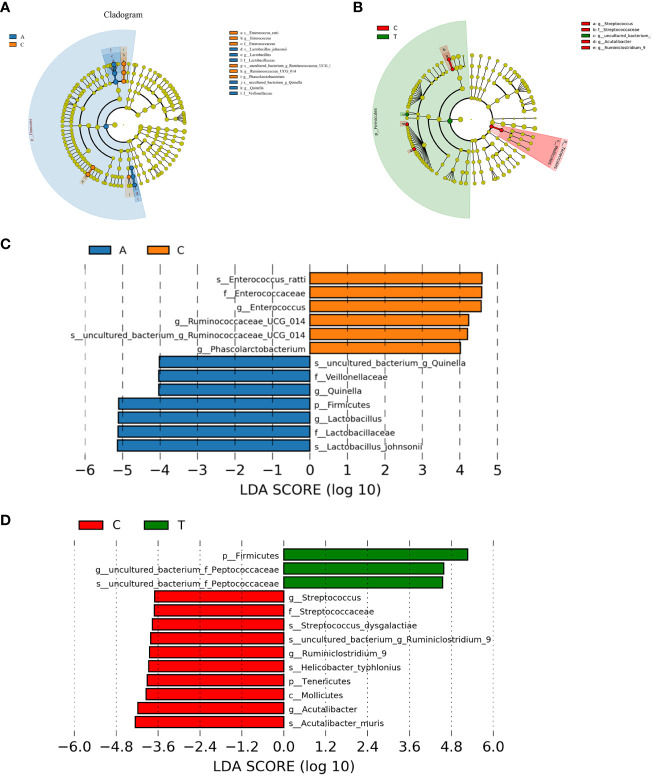
The potential biomarkers were defined based on effect size (LEfSe) combined with linear discriminant analysis (LDA). Cladogram for taxonomic representation of significant differences between Baseline and tMCAO group **(A)** and PSD and PSD+HF-rTMS groups **(B)**. The colored nodes from the inner to the outer circles represent taxa from the phylum to species level. Histogram of the LDA scores for differentially abundant features among groups **(C, D)**. The threshold on the logarithmic LDA score for discriminative features was set to 3.0. A, Baseline group; C, PSD group; T, PSD+HF-rTMS group.

Linear discriminant analysis effect size (LEfSe) was used to identify potential microbiota biomarkers among different groups. *Firmicutes* exhibited a relatively higher abundance at the phylum level in the baseline group than in the PSD group. At the genus level, *Quinella* and *Lactobacillus* were the most abundant at baseline, whereas *Ruminococcaceae_UCG_014*, *Enterococcus*, and *Phascolarctobacterium* were enriched in the PSD group (linear discriminant analysis (LDA) > 3, *p* < 0.05) (**Figures 5A, C**). These microbes might be considered as biomarkers for the Baseline and PSD groups.

At the phylum level, *Firmicutes* was remarkably enriched in the PSD+HF-rTMS group compared to that in the PSD group. At the genus level, the abundance of *uncultured_bacterium_Peptococcaceae* was relatively high in the PSD+HF-rTMS group. The abundance of *Streptococcus*, *Acutalibacter*, and *Ruminiclostridium_9* were relatively high in the PSD group (LDA > 3, *p* < 0.05) (**Figures 5B, D**).

### Pathway analysis of microbiotas

Several KEGG pathways, such as cell growth, cancer-specific types, and immune diseases, were significantly different between the Baseline and PSD group (*p* < 0.05) (**Supplementary Figure 2A**). The pathways in the PSD group and PSD+HF-rTMS group differed in cardiovascular and infectious diseases (viral) (*p* < 0.05) (**Supplementary Figure 2B**).

### Identification of group differential metabolites

**Figure 6:**
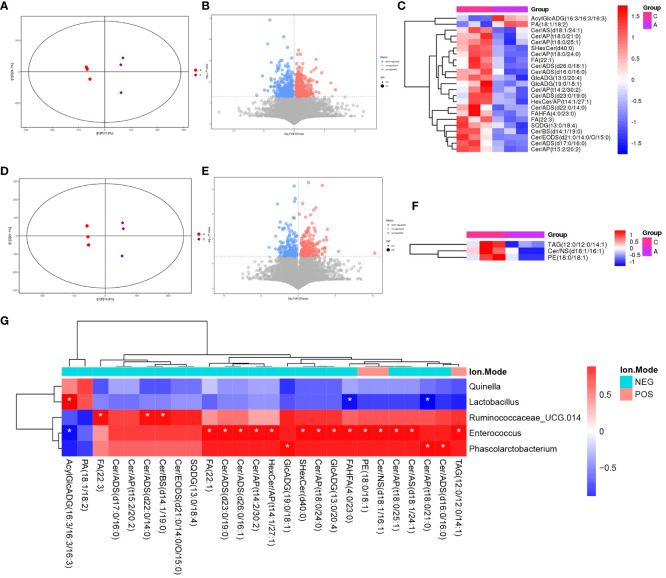
PLS-DA score plots **(A)**, volcano map **(B)**, and heatmap **(C)** of differential metabolites between Baseline and PSD group in the negative ion patterns. PLS-DA score plots **(D)**, volcano map **(E)**, and heatmap **(F)** of differential metabolites in the positive ion patterns. Heatmap of correlation **(G)** between the differential gut microbes and differential metabolites between Baseline and PSD group. NES, Negative Ion Mode; POS, Positive Ion Mode; A, Baseline group; C, PSD group; T, PSD+HF-rTMS group. * *p* < 0.05.

**Figure 7:**
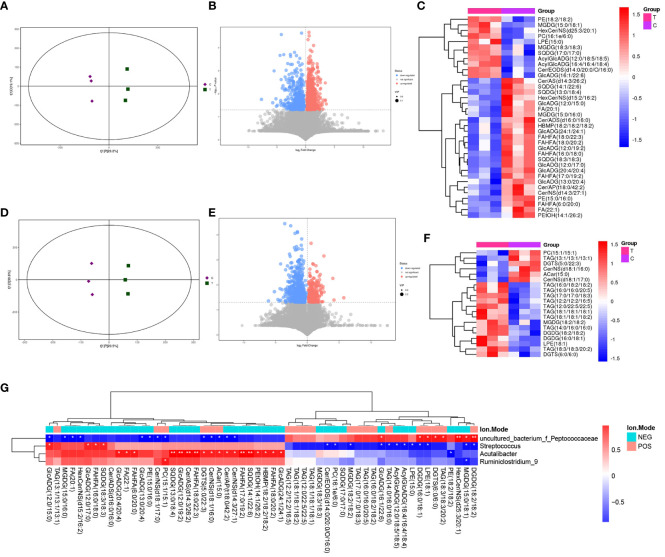
PLS-DA score plots **(A)**, volcano map **(B)**, and heatmap **(C)** of differential metabolites between the PSD+HF-rTMS and PSD group in the negative ion patterns. PLS-DA score plots **(D)**, volcano map **(E)**, and heatmap **(F)** of differential metabolites in the positive ion patterns. Heatmap of correlation **(G)** between the differential gut microbes and differential metabolites between PSD+HF-rTMS and PSD group. NES, Negative Ion Mode; POS, Positive Ion Mode; A, Baseline group; C, PSD group; T, PSD+HF-rTMS group. * *p* < 0.05, ** *p* < 0.01.

LC-MS/MS was used to determine the metabolic characteristics of rats in the three groups. OPLS-DA models showed that the metabolic profile of the PSD group was significantly different from that of the baseline group in both the negative and positive ion modes (**Figures 6A, D**). Similarly, the PSD+HF-rTMS group was significantly different from the PSD group (**Figures 7A, D**).

Differential metabolites were identified using VIP > 1 and *p* < 0.05. A total of 699 differential metabolites [357 upregulated and 342 downregulated]) were screened and identified in the PSD group compared with those in the baseline group in the negative mode (**Figure 6B**). In **Figure 6C**, the heatmap displays a subset of 17 named metabolites (15 upregulated and two downregulated) out of the 699 differential metabolites analyzed. Additionally, in positive-mode analysis (**Figure 6E**), 403 metabolites (234 upregulated and 169 downregulated) were identified and compared. Furthermore, among the 403 differentially expressed metabolites, three upregulated metabolites were visualized with their names in the form of a heat map, as presented in **Figure 6F**. Briefly, the expression levels of differential metabolites in lipid classes, such as ceramides (Cer), free fatty acids (FA), phosphatidylethanolamine (PE), triacylglycerol (TAG), and sulfoquinovosyl diacylglycerol, were increased in PSD.

In the negative mode, a comprehensive analysis of the PSD+HF-rTMS group compared to the PSD group identified 1470 differential metabolites (963 upregulated and 507 downregulated) (**Figure 7B**). Among these 1470 metabolites, 26 specifically named metabolites (11 upregulated and 14 downregulated) were selected for visualization in the form of a heat map (**Figure 7C**). Additionally, in the positive-mode analysis, 919 metabolites (260 upregulated and 659 downregulated) were identified (**Figure 7E**). **Figure 7F** presents a heatmap that displays 20 named metabolites (11 upregulated and 15 downregulated) from the total pool of 919 differential metabolites analyzed. Briefly, the levels of the differential lipids Cer, FA, and DG decreased after HF-rTMS treatment, whereas TAG levels increased.

### Analysis of metabolic pathways

**Figure 8:**
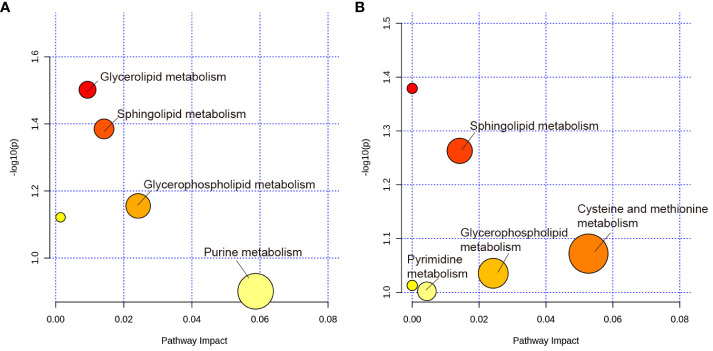
KEGG pathway analysis of group differential metabolites. **(A)** KEGG pathway enrichment analysis of group differential metabolites between the Baseline and PSD group. **(B)** Analysis of group differential metabolites between the PSD+HF-rTMS and PSD group. The metabolic pathways are displayed as distinctly colored circles depending on their enrichment analysis scores (vertical axis, shade of red) and topology (pathway impact, horizontal axis, circle diameter) using MetaboAnalyst 5.0.

The MetaboAnalyst 5.0 was used for MetaboAnalyst pathway analysis of group differential metabolites, with *p* < 0.05. As shown in **Figure 8A**, metabolic pathways, such as glycerolipid, sphingolipid, glycerophospholipid, and urine metabolism, were altered in rats with PSD. HF-rTMS induced changes in sphingolipid, cysteine, methionine, glycerophospholipid, and pyrimidine metabolism in the PSD+HF-rTMS group (**Figure 8B**).

### Correlations between the gut microbiome biomarkers and differential metabolites

Spearman correlation analysis was performed to explore the association between gut microbiota biomarkers and the aforementioned group of differential metabolites (including negative and positive modes). At the genus level, a relationship between the four genera and 21 metabolites was observed between the PSD and Baseline groups (**Figure 6G**). Specifically, Enterococcus was positively correlated with Cer, FA, and PE in the PSD rats. Similarly, a relationship between the four genera and 45 metabolites was observed between the PSD+HF-rTMS and PSD groups. Peptococcaceae was negatively correlated with Cer, Streptococcus was negatively correlated with DG, and Acutalibacter was positively correlated with FA and Cer (**Figure 7G**).

## Discussion

Using VFSS, we found that performing HF-rTMS on the M1 cortex of the unaffected hemisphere for one week can improve swallowing function in PSD rats. This is especially true for bolus area and pharyngeal bolus speed. We observed that PSD rats exhibited damaged surface epithelial cells, disrupted cryptal glands in the colon, and altered intestinal microbiota and associated metabolites, which were ameliorated by the HF-rTMS intervention. These findings highlight the effects of HF-rTMS on the gut microbiota and its metabolites, which are associated with its therapeutic effects.

Numerous studies have demonstrated the efficacy of rTMS in enhancing swallowing function. Both HF-rTMS and LF-rTMS interventions have shown positive outcomes in various studies targeting the ipsilateral or contralateral cerebral cortex of lesion (Cheng et al., 2015; Lee et al., 2015; Park et al., 2017; Du et al., 2022; Hammad et al., 2022). Park et al. (Park et al., 2013) proved that HF-rTMS (5 Hz) over the pharyngeal motor cortex of the unaffected hemisphere significantly improved swallowing function, which is consistent with our findings. This mechanism may involve enhanced stimulation of bulbar motor neurons projecting to the pharynx and further increase corticobulbar excitability (Park et al., 2013). A recent study (Aizawa et al., 2021) suggested that stimulation of the dorsolateral prefrontal cortex by rTMS modifies brain-gut interactions in humans. A comprehensive study has indicated that stroke has the potential to elevate intestinal permeability and activate the intestinal immune system, thus exacerbating ischemia-reperfusion injury through the brain-gut axis. However, some metabolites produced by the intestinal flora attenuate ischemia-reperfusion injury by suppressing the post-stroke inflammatory response and promoting the repair of neurological function through the axis (Hu et al., 2022). Using 16S sequencing, we found that HF-rTMS promoted the restoration of intestinal flora balance. Therefore, after indirect excitatory stimulation, the vagus nerve in the medulla may play an active role via brain-gut interactions.

Using 16S rRNA gene sequencing, we observed that although no significant differences in gut microbiota composition at the phylum level were observed among the three groups, the abundance of the phylum *Firmicutes* was significantly decreased in the PSD group compared with that at baseline. This finding was consistent with the results of previous studies, both in a rat model and in patients (Li et al., 2020; Haak et al., 2021; Wu et al., 2021). *Firmicutes* are the predominant phylum in the intestinal microbiota and contribute to the maintenance of the normal gastrointestinal tract (Ley et al., 2006). The results showed that HF-rTMS increased the abundance of *Firmicutes* to help restore gut microbiota balance. Similarly, Seewoo et al. (Seewoo et al., 2022) found that rTMS (10HZ) is primarily responsible for maintaining a high abundance of *Firmicutes* in a rat model of depression. The abundance of the genus Lactobacillus (phylum Firmicutes), an important type of host probiotic bacterium, was considerably affected in the PSD group. Chen et al. (Chen et al., 2019b) found that the level of *Lactobacillus* decreased in monkeys after MCAO, which was consistent with our findings. *Lactobacillus* can produce SCFAs such as butyrate and acetate, which can improve wound healing, reinforce intestinal epithelial cell integrity in mice with stroke, and inhibit bacterial migration (Cushing et al., 2015; Zou et al., 2022). After HF-rTMS treatment, the relative abundance of *Lactobacillus* showed an upward trend, but did not reach statistical significance, which may be related to the treatment time; prolonging the treatment time may be a better choice. Moreover, H&E staining results further substantiated the effectiveness of HF-rTMS in reinforcing intestinal epithelial cell integrity, which may be correlated with SCFAs. The LEfSe analysis indicated that *Ruminococcaceae_UCG_014*, *Enterococcus*, and *Phascolarctobacterium* were enriched in the PSD group. *Ruminococcaceae_UCG_014* and *Phascolarctobacterium* can produce SCFAs including acetate and propionate (Wu et al., 2017; Tian et al., 2021). Their enrichment in the PSD group may be related to a compensatory response secondary to the massive reduction in the level of SCFA-producing bacteria, especially butyrate-producing bacteria such as *Lactobacillus*. *Enterococcus* is an opportunistic pathogen that can cause pneumonia and bacteremia in patients with stroke, resulting in unfavorable outcomes even 3 month post-stoke (Stanley et al., 2016; Sun et al., 2021). Furthermore, LEfSe analysis showed that the abundance of *uncultured_bacterium_Peptococcaceae* was relatively high after HF-rTMS treatment, whereas *Streptococcus*, *Acutalibacter*, and *Ruminiclostridium_9* were enriched in the PSD group. *Peptococcaceae* and *Ruminiclostridium_9* are also groups of bacteria characterized by the production of SCFA after the degradation of indigestible plant-derived polysaccharides (Bernad-Roche et al., 2021). *Streptococcus* and *Acutalibacter* are proinflammatory bacteria. PSD increased the levels of opportunistic pathogens and decreased the levels of SCFA-producing bacteria, particularly *Lactobacillus*, whereas HF-rTMS increased SCFA-producing bacteria and decreased pro-inflammatory bacteria levels. Chen et al. demonstrated that interfering with the gut microbiota by transplanting SCFA-rich fecal bacteria is an effective treatment for cerebral ischemic stroke (Chen et al., 2019a). Thus, HF-rTMS may enhances brain-gut interactions via SCFA to effectively improve PSD swallowing function, and the effect was similar to that of transplanting feces.

Combined with untargeted metabolomic analysis, we found that PSD was associated with disturbances in fecal metabolomics. Pathway analysis showed that these metabolites are primarily involved in glycerolipid, sphingolipid, glycerophospholipid, and urine metabolism. A previous study has shown that stroke is related to urine and sphingolipid metabolism (Ding et al., 2022). Furthermore, the results indicated that sphingolipid metabolism, cysteine and methionine metabolism, glycerophospholipid metabolism, and pyrimidine metabolism are potential target metabolic pathways of HF-rTMS. Moreover, we investigated the association between gut microbiota and fecal metabolites. This analysis showed that *Enterococcus* was positively correlated with Cer, FA, and PE levels in the PSD rats. As these levels were all significantly increased in the PSD group, they may be risk factors for PSD. After administration of HF-rTMS, *Peptococcaceae* was negatively correlated with Cer, *Streptococcus* was negatively correlated with DG, *Acutalibacter* was positively correlated with FA and Cer. These untargeted metabolomics findings suggest that HF-rTMS has the potential to induce modifications in the composition of the gut microbiota, thus influencing the levels of fecal metabolites. These alterations in metabolites are expected to affect specific metabolic pathways, ultimately resulting in therapeutic effects in PSD.

In summary, our research findings validate that HF-rTMS can effectively ameliorate intestinal flora imbalance and reinstate the integrity of the intestinal wall. This restoration may potentially mitigate intestinal inflammation and diminish the influx of toxins into the bloodstream, thus alleviating cerebral ischemia injury. Additionally, HF-rTMS augments the population of SCFAs-producing bacteria, leading to the production of metabolites such as SCFAs. These metabolites exhibit a protective role against cerebral ischemic injury and facilitate the functional recovery of PSD by curbing inflammation, restraining bacterial migration, among other mechanisms (Cushing et al., 2015; Zou et al., 2022). Presently, a multitude of animal models predominantly utilize fecal microbiota transplantation (FMT) to rectify intestinal flora imbalances. This approach proves to be financially burdensome in clinical settings and is not readily available in general hospital settings. Furthermore, FMT encounters formidable hurdles stemming from the potential transmission of diseases between donors and recipients, patient reluctance, adverse repercussions, and the ambiguity surrounding its impact on the recipient’s immune system, encompassing conditions such as peripheral neuropathy and idiopathic thrombocytopenic purpura (Wang et al., 2019; Porcari et al., 2023). rTMS therapy, a non-invasive treatment that does not require the special cooperation of the patient, which can be used as an alternative therapy to bacterial transplantation, improves intestinal flora disorders through the brain-gut axis and in turn promotes stroke rehabilitation.

This study had some limitations. Although we propose a potential contribution of HF-rTMS to the gut microbiome and fecal metabolites, the mechanisms underlying the gut-brain interactions remain unclear and require further elucidation. Therefore, extensive evaluation of additional metabolites, including neurotransmitters and SCFAs, is crucial for establishing the comprehensive efficacy of HF-rTMS. Prior investigations (Aizawa et al., 2021; Seewoo et al., 2022) have highlighted the impact of HF-rTMS on the gastrointestinal system. Notably, in our study, we observed a significant increase in rat defecation rates following HF-rTMS stimulation. We hypothesize that the application of high-frequency transcranial magnetic stimulation to the M1 region in rat models could activate the dorsal vagal nucleus situated within the medulla oblongata. This activation, in turn, may enhance gastrointestinal motility via the vagus nerve, potentially mitigating dysbiosis. So subsequent studies will focus on assessing the role of the dorsal vagal complex and vagus nerve in the therapeutic mechanism of HF-rTMS. Understanding the involvement of the dorsal vagal complex-vagus nerve-gut axis holds a tempting prospect for advancing our knowledge in this field. Finally, further clinical research is warranted to validate our findings.

## Conclusion

In conclusion, we employed a combination of intestinal pathology, 16S rRNA gene sequencing, and LC-MS analysis to compare the colonic structure, composition, and abundance of gut microbiota and fecal metabolites among three groups: Baseline, PSD, and PSD with HF-rTMS treatment. We observed that PSD rats exhibited damaged surface epithelial cells and disrupted cryptal glands in the colon, which were ameliorated by HF-rTMS. Our investigation of the gut microbiota revealed a significant decrease in the presence of SCFA-producing bacteria in rats with PSD, with notable augmentation following the HF-rTMS intervention. Moreover, using fecal metabolomic analysis, we identified distinct metabolites that exhibited variation across groups. Finally, our integrated analysis demonstrated a correlation between the gut microbiota and dysregulated metabolites. These findings suggest a synergistic role for the gut microbiota and fecal metabolites in the development of PSD and the therapeutic mechanisms underlying HF-rTMS. This mechanism may be related to changes in the brain-gut axis and warrants further investigation to fully understand and use HF-rTMS for treating PSD.

## Data availability statement

The data presented in the study are deposited in the National Center for Biotechnology Information (NCBI) repository, accession number PRJNA1086134.

## Ethics statement

All animal experiments were approved by the Animal Care Committee of the South China Agricultural University (Guangzhou, China) and were performed in accordance with the Guidelines for the Care and Use of Laboratory Animals issued by the Chinese Council on Animal Research. The study was conducted in accordance with the local legislation and institutional requirements.

## Author contributions

FZ: Conceptualization, Data curation, Formal analysis, Writing – original draft, Writing – review & editing. JC: Conceptualization, Data curation, Methodology, Writing – original draft. YS: Conceptualization, Methodology, Data curation, Writing – original draft. JNH: Methodology, Writing – original draft. QY: Methodology, Writing – original draft. YD: Methodology, Software, Writing – original draft. JHH: Methodology, Software, Writing – original draft. JZ: Methodology, Writing – original draft. CL: Funding acquisition, Project administration, Writing – original draft. HW: Funding acquisition, Supervision, Conceptualization, Writing – review & editing.

## Glossary

**Table:**

Acar	Acylcarnitine
AcylGlcADG	Acylglucuronosyldiacylglycerol
Cer/ADS	Ceramide alpha-hydroxy fatty aciddihydrosphingosine
Cer/AP	Ceramide alpha-hydroxy fatty acidphytospingosine
Cer/AS	Ceramide alpha-hydroxy fatty acid-sphingosine
Cer/ BS	Ceramide beta-hydroxy fatty acid-sphingosine
Cer/EODS	Ceramide Esterified omega-hydroxy fatty acid-dihydrosphingosine
Cer/NS	Ceramide non-hydroxyfatty acid-sphingosine
DGDG	Digalactosyldiacylglycerol
DGTS	Diacylglyceryl trimethylhomoserine
FA	Free fatty acid
FAHFA	Fatty acid ester of hydroxyl fatty acid
GlcADG	glucuronosyldiacylglycerol
HBMP	Hemibismonoacylglycerophosphate
HexCer/AP	Hexosylceramide alphahydroxy fatty acid-phytospingosine
HexCer/NS	Hexosylceramide nonhydroxyfatty acid-sphingosine
HF-TMS	High frequency repetitive transcranial magnetic stimulation
ISI	inter-swallow interval
LEfSe	Linear discriminant analysis Effect Size
LF-rTMS	Low frequency repetitive transcranial magnetic stimulation
LPE	Lysophosphatidylethanolamine
M1	Primary motor cortex
MGDG	Monogalactosyldiacylglycerol
NSS	neurological severity scores
OPLSDA	Orthogonal Projections to Latent Structures Discriminant Analysis
OTUs	operational taxonomic units
PA	Phosphatidic acid
PC	Phosphatidylcholine
PCA	Principal component analysis
PCoA	principal coordinate analysis
PE	Phosphatidylethanolamine
PEtOH	Phosphatidylethanol
PSD	post-stroke dysphagia
PTT	pharyngeal transit time
SCFAs	short-chain fatty acids
SHexCer	SulfurHexosylceramide hydroxyfatty acid
SQDG	Sulfoquinovosyl diacylglycerol
TAG	triacylglycerol
tMCAO	transient middle cerebral artery occlusion
VFSS	Videofluoroscopy swallowing study

## References

[B1] Aizawa Y. Morishita J. Kano M. Kanazawa M. Fukudo S. (2021). Modification of rectal function and emotion by repetitive transcranial magnetic stimulation in humans. Neurosci. Res. 168, 54–63. doi: 10.1016/j.neures.2021.05.013 34062217

[B2] Benakis C. Martin-Gallausiaux C. Trezzi J. Melton P. Liesz A. Wilmes P. (2020). The microbiome-gut-brain axis in acute and chronic brain diseases. J. Mol. Neurosci. 61, 1–9. doi: 10.1016/j.conb.2019.11.009 31812830

[B3] Bernad-Roche M. Bellés A. Grasa L. Casanova-Higes A. Mainar-Jaime R. (2021). Effects of dietary supplementation with protected sodium butyrate on gut microbiota in growing-finishing pigs. Anim. (Basel). 11(7):2137. doi: 10.3390/ani11072137 PMC8300649 34359264

[B4] Bonilha H. Simpson A. Ellis C. Mauldin P. Martin-Harris B. Simpson K. (2014). The one-year attributable cost of post-stroke dysphagia. Dysphagia 29, 545–552. doi: 10.1007/s00455-014-9543-8 24948438 PMC4179977

[B5] Chen Y. Liang J. Ouyang F. Chen X. Lu T. Jiang Z.. (2019b). Persistence of gut microbiota dysbiosis and chronic systemic inflammation after cerebral infarction in cynomolgus monkeys. Front. Neurol. 10. doi: 10.3389/fneur.2019.00661 PMC6611357 31316450

[B6] Chen R. Xu Y. Wu P. Zhou H. Lasanajak Y. Fang Y.. (2019a). Transplantation of fecal microbiota rich in short chain fatty acids and butyric acid treat cerebral ischemic stroke by regulating gut microbiota. Pharmacol. Res. 148, 104403. doi: 10.1016/j.phrs.2019.104403 31425750

[B7] Cheng I. Chan K. Wong C. Cheung R. (2015). Preliminary evidence of the effects of high-frequency repetitive transcranial magnetic stimulation (rTMS) on swallowing functions in post-stroke individuals with chronic dysphagia. Int. J. Lang Commun. Disord. 50, 389–396. doi: 10.1111/1460-6984.12144 25588767

[B8] Chiang C. Lin M. Hsiao M. Yeh Y. Liang Y. Wang T. (2019). Comparative efficacy of noninvasive neurostimulation therapies for acute and subacute poststroke dysphagia: A systematic review and network meta-analysis. Arch. Phys. Med. Rehabil. 100, 739–750.e734. doi: 10.1016/j.apmr.2018.09.117 30352222

[B9] Cushing K. Alvarado D. Ciorba M. (2015). Butyrate and mucosal inflammation: new scientific evidence supports clinical observation. Clin. Transl. Gastroenterol. 6, e108. doi: 10.1038/ctg.2015.34 26312412 PMC4816278

[B10] Dawson J. Pierce D. Dixit A. Kimberley T. Robertson M. Tarver B.. (2016). Safety, feasibility, and efficacy of vagus nerve stimulation paired with upper-limb rehabilitation after ischemic stroke. Stroke 47, 143–150. doi: 10.1161/STROKEAHA.115.010477 26645257 PMC4689175

[B11] Ding X. Liu Z. Liu Y. Xu B. Chen J. Pu J.. (2022). Comprehensive evaluation of the mechanism of Blume in ameliorating cerebral ischemia-reperfusion injury based on integrating fecal metabonomics and 16S rDNA sequencing. Front. Cell Infect. Microbiol. 12. doi: 10.3389/fcimb.2022.1026627 PMC9648199 36389137

[B12] Du Y. Wei L. Lu Y. Gao H. (2022). The effects of different frequencies of repetitive transcranial magnetic stimulation (rTMS) on patients with swallowing disorders after cerebral infarction. NeuroRehabilitation 50, 115–122. doi: 10.3233/NRE-210176 34776422

[B13] Haak B. Westendorp W. van Engelen T. Brands X. Brouwer M. Vermeij J.. (2021). Disruptions of anaerobic gut bacteria are associated with stroke and post-stroke infection: a prospective case-control study. Transl. Stroke Res. 12, 581–592. doi: 10.1007/s12975-020-00863-4 33052545 PMC8213601

[B14] Hammad A. Elhamrawy E. Abdel-Tawab H. Shafik M. Sallam Y. Elzomor H.. (2022). Transcranial magnetic stimulation versus transcutaneous neuromuscular electrical stimulation in post stroke dysphagia: A clinical randomized controlled trial. J. Stroke Cerebrovasc. Dis. 31, 106554. doi: 10.1016/j.jstrokecerebrovasdis.2022.106554 35691184

[B15] Hu W. Kong X. Wang H. Li Y. Luo Y. (2022). Ischemic stroke and intestinal flora: an insight into brain-gut axis. Eur. J. Med. Res. 27, 73. doi: 10.1186/s40001-022-00691-2 35614480 PMC9131669

[B16] Khedr E. M. Abo-Elfetoh N. (2009). Treatment of post-stroke dysphagia with repetitive transcranial magnetic stimulation. Acta Neurol. Scand. 119, 155–161. doi: 10.1111/ane.2009.119.issue-3 18771521

[B17] Lee J. Kim S. Lee K. Lee S. Lee J. (2015). Effect of repetitive transcranial magnetic stimulation according to the stimulation site in stroke patients with dysphagia. Ann. Rehabil. Med. 39, 432–439. doi: 10.5535/arm.2015.39.3.432 26161350 PMC4496515

[B18] Ley R. Peterson D. Gordon J. (2006). Ecological and evolutionary forces shaping microbial diversity in the human intestine. Cell 124, 837–848. doi: 10.1016/j.cell.2006.02.017 16497592

[B19] Li H. Zhang X. Pan D. Liu Y. Yan X. Tang Y.. (2020). Dysbiosis characteristics of gut microbiota in cerebral infarction patients. Transl. Neurosci. 11, 124–133. doi: 10.1515/tnsci-2020-0117 33312718 PMC7706127

[B20] Liao X. Xing G. Guo Z. Jin Y. Tang Q. He B.. (2017). Repetitive transcranial magnetic stimulation as an alternative therapy for dysphagia after stroke: a systematic review and meta-analysis. Clin. Rehabil. 31, 289–298. doi: 10.1177/0269215516644771 27113337

[B21] Lin C. Chung S. Lin C. Hwu Y. (2021). Effect of tongue-to-palate resistance training on tongue strength in healthy adults. Auris Nasus Larynx. 48, 116–123. doi: 10.1016/j.anl.2020.07.014 32727703

[B22] Park E. Kim M. Chang W. Oh S. Kim Y. Lee A.. (2017). Effects of bilateral repetitive transcranial magnetic stimulation on post-stroke dysphagia. rain Stimul. 10, 75–82. doi: 10.1016/j.brs.2016.08.005 27593709

[B23] Park J. Oh J. Lee J. Yeo J. Ryu K. (2013). The effect of 5Hz high-frequency rTMS over contralesional pharyngeal motor cortex in post-stroke oropharyngeal dysphagia: a randomized controlled study. Neurogastroenterol Motil. 25, 324–e250. doi: 10.1111/nmo.12063 23279198

[B24] Porcari S. Benech N. Valles-Colomer M. Segata N. Gasbarrini A. Cammarota G.. (2023). Key determinants of success in fecal microbiota transplantation: From microbiome to clinic. Cell Host Microbe 31, 712–733. doi: 10.1016/j.chom.2023.03.020 37167953

[B25] Reyes-Torres C. Castillo-Martínez L. Reyes-Guerrero R. Ramos-Vázquez A. Zavala-Solares M. Cassis-Nosthas L.. (2019). Design and implementation of modified-texture diet in older adults with oropharyngeal dysphagia: a randomized controlled trial. Eur. J. Clin. Nutr. 73, 989–996. doi: 10.1038/s41430-019-0389-x 30643223

[B26] Russell J. Ciucci M. Hammer M. Connor N. (2013). Videofluorographic assessment of deglutitive behaviors in a rat model of aging and Parkinson disease. Dysphagia 28, 95–104. doi: 10.1007/s00455-012-9417-x 22763806 PMC3554861

[B27] Seewoo B. Chua E. Arena-Foster Y. Hennessy L. Gorecki A. Anderton R.. (2022). Changes in the rodent gut microbiome following chronic restraint stress and low-intensity rTMS. Neurobiol. Stress 17, 100430. doi: 10.1016/j.ynstr.2022.100430 35146078 PMC8819474

[B28] Shigematsu T. Fujishima I. (2015). Dysphagia and swallowing rehabilitation. Brain Nerve. 67, 169–182. doi: 10.11477/mf.1416200109 25681362

[B29] Singh V. Roth S. Llovera G. Sadler R. Garzetti D. Stecher B.. (2016). Microbiota dysbiosis controls the neuroinflammatory response after stroke. J. Neurosci. 36, 7428–7440. doi: 10.1523/JNEUROSCI.1114-16.2016 27413153 PMC6705544

[B30] Stanley D. Mason L. Mackin K. Srikhanta Y. Lyras D. Prakash M.. (2016). Translocation and dissemination of commensal bacteria in post-stroke infection. Nat. Med. 22, 1277–1284. doi: 10.1038/nm.4194 27694934

[B31] Sun H. Gu M. Li Z. Chen X. Zhou J. (2021). Gut microbiota dysbiosis in acute ischemic stroke associated with 3-month unfavorable outcome. Front. Neurol. 12. doi: 10.3389/fneur.2021.799222 PMC8831883 35153980

[B32] Tian B. Zhao J. Zhang M. Chen Z. Ma Q. Liu H.. (2021). Lycium ruthenicum anthocyanins attenuate high-fat diet-induced colonic barrier dysfunction and inflammation in mice by modulating the gut microbiota. Mol. Nutr. Food Res. 65, e2000745. doi: 10.1002/mnfr.202000745 33629483

[B33] Wang J.-W. Kuo C.-H. Kuo F.-C. Wang Y.-K. Hsu W.-H. Yu F.-J.. (2019). Fecal microbiota transplantation: Review and update. J. Formos Med. Assoc. 118 (1), S23–S31. doi: 10.1016/j.jfma.2018.08.011 30181015

[B34] Welby L. Ukatu C. Thombs L. Lever T. (2021). A mouse model of dysphagia after facial nerve injury. Laryngoscope 131, 17–24. doi: 10.1002/lary.28560 32096879

[B35] Wu F. Guo X. Zhang J. Zhang M. Ou Z. Peng Y. (2017). Phascolarctobacterium faecium abundant colonization in human gastrointestinal tract. Exp. Ther. Med. 14, 3122–3126. doi: 10.3892/etm.2017.4878 28912861 PMC5585883

[B36] Wu W. Sun Y. Luo N. Cheng C. Jiang C. Yu Q.. (2021). Integrated 16S rRNA gene sequencing and LC-MS analysis revealed the interplay between gut microbiota and plasma metabolites in rats with ischemic stroke. J. Mol. Neurosci. 71, 2095–2106. doi: 10.1007/s12031-021-01828-4 33954858

[B37] Zhang M. Li C. Zhang F. Han X. Yang Q. Lin T.. (2021). Prevalence of dysphagia in China: an epidemiological survey of 5943 participants. Dysphagia 36, 339–350. doi: 10.1007/s00455-020-10138-7 32458145

[B38] Zhong Y. Gu L. Ye Y. Zhu H. Pu B. Wang J.. (2022). JAK2/STAT3 axis intermediates microglia/macrophage polarization during cerebral ischemia/reperfusion injury. Neuroscience 496, 119–128. doi: 10.1016/j.neuroscience.2022.05.016 35598702

[B39] Zou X. Wang L. Xiao L. Wang S. Zhang L. (2022). Gut microbes in cerebrovascular diseases: Gut flora imbalance, potential impact mechanisms and promising treatment strategies. Front. Immunol. 13. doi: 10.3389/fimmu.2022.975921 PMC9659965 36389714

