# Supplementary material for: Comprehensive assessment of HF-rTMS treatment mechanism for post-stroke dysphagia in rats by integration of fecal metabolomics and 16S rRNA sequencing
Source: Front Cell Infect Microbiol. 2024 Apr 15;14:1373737. doi: 10.3389/fcimb.2024.1373737 (PMC11057012; doi:10.3389/fcimb.2024.1373737)
Supplement: Supplementary file 1 [file DataSheet_1.docx]

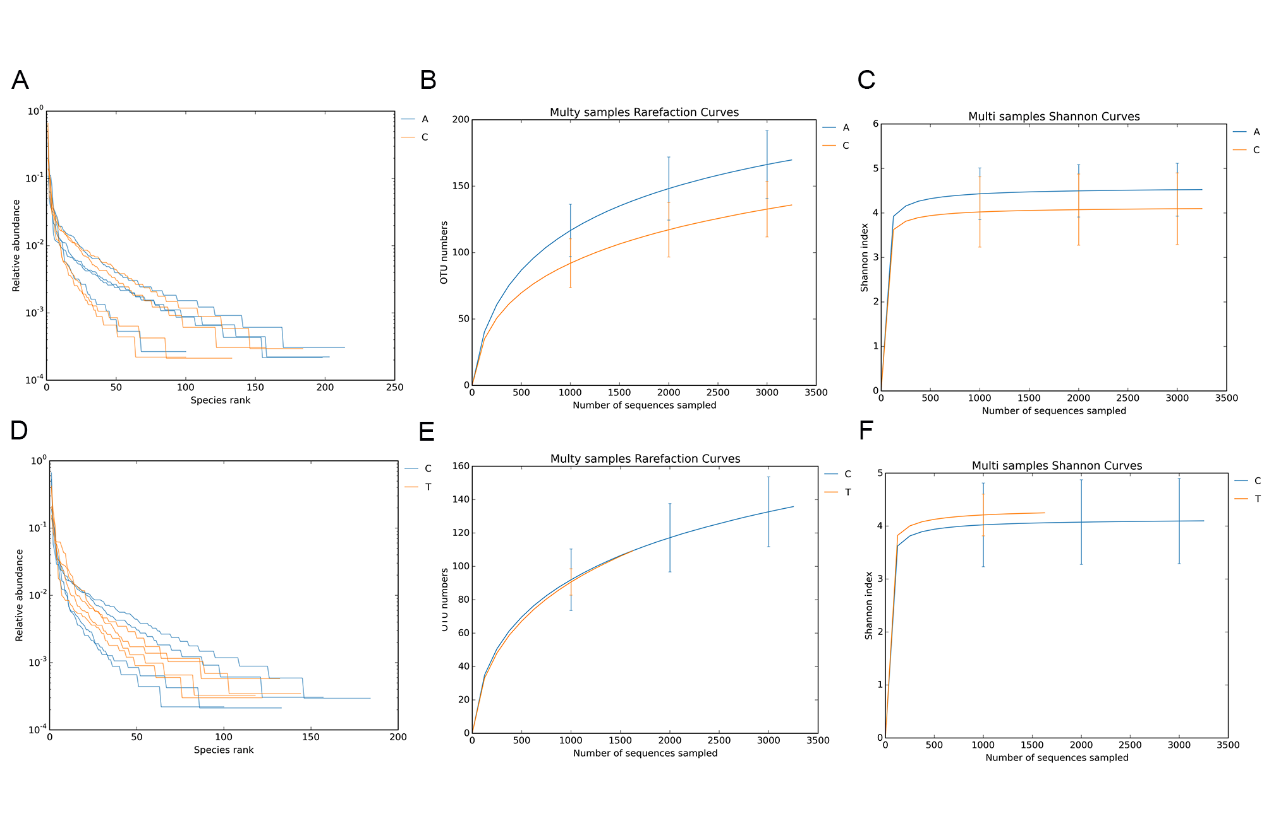
Figure 1. OTU rank-abundance distribution curve (A, D), Rarefaction analysis of the microbiota (B, E) and Shannon-Wiener curve (C, F). A, Baseline group; C, PSD group; T, PSD+HF-rTMS group.


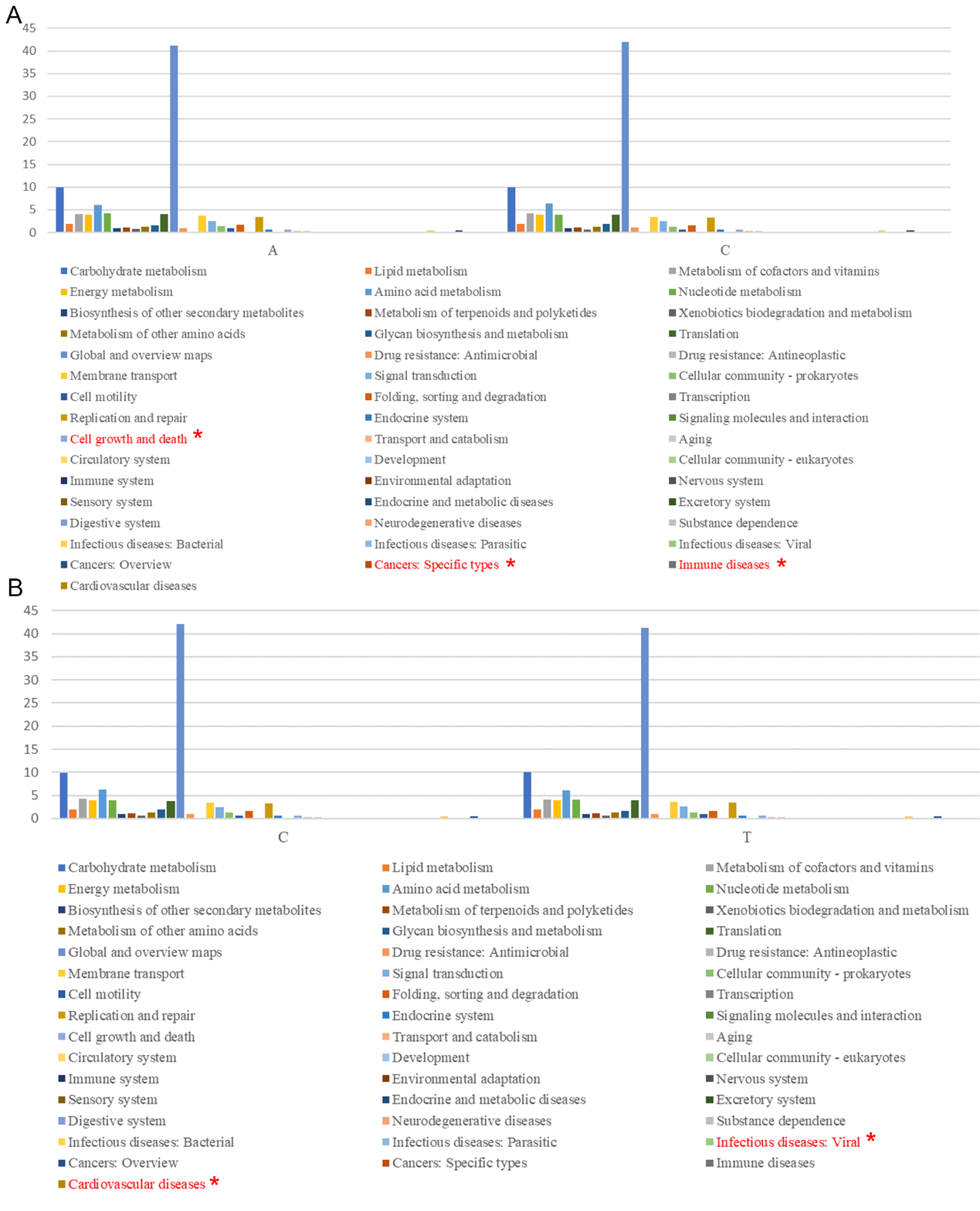
Figure 2. Functional predictive analysis. Microbial community functions against KEGG database between different groups predicted by PICRUSt. The compositions of KEGG class2 function in the baseline vs. PSD groups (A), PSD vs. PSD+HF-rTMS group (B). A, Baseline group; C, PSD group; T, PSD+HF-rTMS group. * *p* < 0.05.
